# Fourier computed tomographic imaging of two dimensional fluorescent objects

**DOI:** 10.1063/1.5100525

**Published:** 2019-10-01

**Authors:** Patrick A. Stockton, Keith A. Wernsing, Jeffrey J. Field, Jeff Squier, Randy A. Bartels

**Affiliations:** 1 Department of Electrical and Computer Engineering, Colorado State University, Ft. Collins, Colorado 80523, USA; 2 Department of Biochemistry and Molecular Biology, Colorado State University, Ft. Collins, Colorado 80523, USA; 3 Microscope Imaging Network Foundational Core Facility, Colorado State University, Ft. Collins, Colorado 80523, USA; 4 Center for Microintegrated Optics for Advanced Biological Control, Department of Physics, Colorado School of Mines, Golden, Colorado 80401, USA; 5 School of Biomedical Engineering, Colorado State University, Ft. Collins, Colorado 80523, USA

## Abstract

We introduce a new form of tomographic imaging that is particularly advantageous for a new class of super-resolution optical imaging methods. Our tomographic method, Fourier Computed Tomography (FCT), operates in a conjugate domain relative to conventional computed tomography techniques. FCT is the first optical tomography method that records complex projections of the object spatial frequency distribution. From these spatial frequency projections, the spatial slice theorem is derived, which is used to build a tomographic imaging reconstruction algorithm. FCT enables enhancement of spatial frequency support along a single spatial direction to be isotropic in the entire transverse spatial frequency domain.

## I. INTRODUCTION

Optical microscopy is an indispensable tool in many fields of science. A major advantage is that visible light is gentle on biological specimens and, in combination with fluorescent labeling, gives high contrast images with excellent specificity. Information recorded in an imaging system is limited by constraints of optical wavelength, propagation, and detection geometry. These constraints combine to limit the recorded information to a subset of the total information potentially available to fully describe the object. Complete object information can be obtained from a sequence of measurements designed to add diversity in spatial or spatial frequency domains. The recorded information forms a set of measurements from which an image is obtained that more closely represents the true object. Imaging systems that expand the recorded information through a sequence of measurements are known as tomographic imaging methods. These methods measure a set of projections in a low-spatial dimension to recover objects in a higher-spatial dimension. The classic method of computed tomography (CT) uses x-ray illumination to measure the object’s integrated absorption along the x-ray beam propagation direction. High quality spatial maps of x-ray absorption or object density can be reconstructed from a set of projections measured at distinct angles.^1,2^

Tomography has been extended to visible light by taking into account photophysics such as optical diffraction.^1,3^ Each tomographic image reconstruction strategy exploits an understanding of the underlying physics, from which an image projection model is constructed. A wide variety of tomographic imaging methods have emerged that provide detailed object information from a range of measurements, such as backscattered light gated by low-coherence interferometry for optical coherence tomography (OCT),^3,4^ diffraction tomographic microscopy,^5^ the diffuse propagation of light in tissues,^6,7^ phase nanoscopy,^8^ fluorescent optical projection tomography,^9,10^ white light diffraction tomography,^11^ limited angle quantitative phase tomography,^12^ and forward scattered light.^13^

Many of these optical tomographic imaging systems use a camera-based optical microscope to form images while the object is rotated or translated along the optic axis or the illumination angle is swept. While modern cameras are well developed and provide exceptional imaging quality and speed, in applications where the sample exhibits optical scattering, the illumination light and recorded images are significantly distorted.^14^ Single pixel imaging methods, such as confocal microscopy, multiphoton, and OCT, are able to perform robust imaging in scattering media; however, all of these methods record one spatial point at a time, and the sequential image acquisition significantly slows image acquisition rates.

To address this limitation, we have developed several single pixel optical tomographic imaging techniques based on mapping spatial position to distinct temporal modulation frequencies of illumination light.^15–18^ These techniques fall into a class of imaging methods called Spatial Frequency Projection (SFP) imaging, where images are formed by a series of spatial frequency projections enabled by periodically modulated illumination light. SFP gives a distinct mapping of temporal modulation frequency to points in space by linearly sweeping through all spatial frequencies supported by the imaging system.

The idea of spatial frequency projections can be extended to super-resolution by driving nonlinear optical interactions in the sample, which we have demonstrated with MultiPhoton SPatIal Frequency Projection Imaging (MP-SPIFI).^19^ MP-SPIFI uses an intense ultrafast laser pulse brought to a line focus that drives a nonlinear optical response in the sample to generate spatial frequency harmonics. The spatial frequency harmonics driven by the nonlinear response produce signals that obtain information from spatial frequencies outside of the diffraction-limited spatial frequency imaging bandwidth, thus allowing super resolution imaging. MP-SPIFI is the only general super-resolution technique that is able to provide super resolution images for both coherent and incoherent imaging modalities.

Although high quality images are produced with MP-SPIFI, to date, the improvements in imaging resolution are limited to one spatial dimension (e.g., *x*). In principle, the full spatial resolution of the SFP imaging process can be extended to the 2D lateral plane (*x*, *y*) by using a previously reported method of lateral tomographic (LT) imaging;^15^ this approach is not practical for MP-SPIFI because LT requires spatial expansion of the illumination beam, reducing the peak illumination intensity and thereby shutting down the nonlinear optical process.

**FIG. 1.**
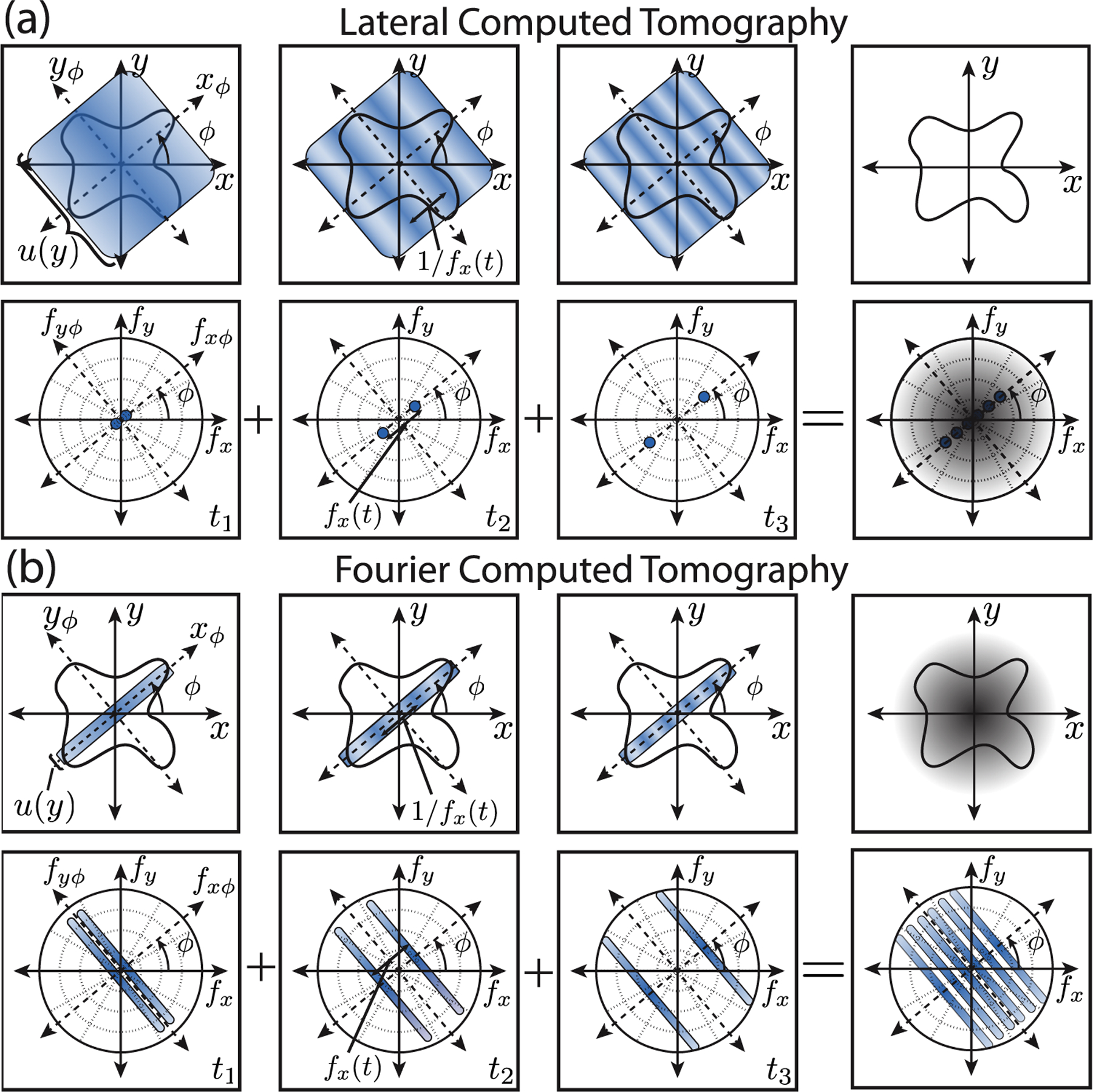
Panel (a) shows the data flow for lateral computed tomography (CT). The top three figures show a series of snapshots in time of the illumination beam and the object at an illumination angle *ϕ*. At each point in time, a unique spatial frequency, *f*_*x*_(*t*) = *κt*, is projected onto the sample. The detection photodiode performs a 2D spatial integration and acts like a Fourier transform, sensing the object in the spatial frequency domain. The second row shows the spatial frequencies probed as a function of time. The projections are summed together and weighted with a radial filter. Rotating the illumination beam 180° maps out the full frequency support of the microscope. An inverse Fourier transform yields a reconstruction of the 2D object. Panel (b) shows the conjugate analog of lateral CT called Fourier CT. The third row shows a series of snapshots in time of the illumination and object at an angle *ϕ*. The bottom row shows the corresponding spatial frequency support probed as a function of time. The frequency support is summed together (bottom right). An inverse Fourier transform is taken and a radial spatial filter is applied to yield a reconstruction of the object.

In this work, we introduce a new 2-dimensional tomographic imaging technique that enables nearly isotropic lateral resolution. This new Fourier Computed Tomography (FCT) technique is based on collecting *spatial frequency projections* of the object in the *x*-*y* plane when the illumination beam is brought to a tight line focus. The method is a conjugate domain analog to computed and lateral tomography techniques. In CT and LT, line integrals along a coordinate (*z*_*ϕ*_ or *y*_*ϕ*_, respectively) are formed in the spatial domain. In the spatial frequency domain, these projections are localized to a point and the sequence of projections form a line at the same rotation angle, *ϕ* [see conceptual diagram Fig. 1(a)]. The formal mathematical description of these projections leads to the Fourier slice theorem.^2^ However, in FCT, the projection operates in the conjugate domain to CT/LT by acquiring line images with SFP illumination in the spatial domain. The tightly focused line in space is equivalent to recording a line projection in the spatial frequency domain for each instantaneous spatial frequency projection, *f*_*x*_(*t*), of the illumination pattern [Fig. 1(b)]. In FCT, we find an analogous spatial slice theorem that motivates the development of a spatial frequency filtered back-projection reconstruction algorithm to produce 2D images from the measured FCT sinograms.

## II. THEORY

The single pixel tomographic imaging process uses an illumination light pattern that imparts a distinct temporal modulation frequency to each spatial position along a line of illumination, *I*_*i*_(*x*, *y*, *t*) = *u*(*y*)[1 + cos(*ω*_*c*_*t* + 2*πκxt*)]^2^. Here, we have suppressed unimportant scaling coefficients, and *u*(*y*) is the intensity profile along the *y* coordinate. Spatiotemporal modulation parameters are set by the carrier frequency, *ω*_*c*_, and the modulation chirp parameter, *κ*, that relates modulation frequency of the illumination light to *x* position as *ω*_*m*_(*x*) = *ω*_*c*_ + 2*πκx*. Light collected from the sample is detected on a photodiode or photomulitplier tube (PMT), and the acquired temporal signal can be written as a spatial projection of the illumination intensity onto the object, *S*_*t*_ = 〈*I*_*i*_(*x*, *y*, *t*)*c*(*x*, *y*)〉_*x*,*y*_. Here, the Dirac integral notation ${\langle \cdot \rangle }_{\bar{v}}=\int d\bar{v}$ denotes the spatial integral performed by a single pixel detector that sums the local photocurrent from light intensity impinging across the detector surface.

In this article, we refer to several types of “projections,” either in the spatial or the spatial frequency domain, where a projection is describing the action of sensing an object in some domain ${\mathbb{R}}^{n}$ and detecting a signal in a lower dimension ${\mathbb{R}}^{n-m}$, where *m* is some integer in the range 0 < *m* < *n*. The object, *c*(*x*, *y*), is assumed to be thin; however, the theory developed below can be extended to optically thick objects.

The object information is collected in the Fourier basis and can be easily isolated in a sideband centered at the carrier frequency, *ω*_*c*_, with a simple discrete Fourier transform applied to the collected signal. In order to develop the theory below, it is convenient to work with the demodulated single sideband,^19^ ${\tilde{S}}_{t}^{\left(q\right)}\;=\;{\langle u(y)c(x,y)e^{-i2\pi q\kappa tx}\rangle }_{x,y}$, where *q* = [1, 2, …] describes the imaging order, *f*_*x*_(*t*) = *κt* is the projected spatial frequency at time, *t*, and *e*^−*i*2*πqκtx*^ is the complex modulation; see conceptual diagram in Fig. 1, which shows the real part of the complex modulation in the top row. Note that *q* scales the effective coherent spatial frequency pass band of the imaging process allowing for lateral resolution enhancement.^17,19^ To observe information corresponding to *q* > 2, intense illumination light can be used to drive nonlinear processes such that the illumination pattern with respect to the sample becomes distorted. The distorted illumination can be described as an effective illumination light pattern, *I*_eff_ = *F*[*I*_*i*_], where *F*[·] is some nonlinear function that models a physical process, such as 2-photon excited fluorescence (2PEF), coherent nonlinear scattering, or saturated absorption. The effective illumination contains modulation frequency harmonics of the temporal modulation, *ω*_*q*_ = *qω*_*c*_. The modulation harmonics encode higher spatial frequency projections allowing for resolution enhancement along the modulated dimension. We have applied this technique to simultaneously acquire super-resolution images of 2PEF and second harmonic generation, i.e., $I_{\text{eff}}\;=\;I_{i}^{2}$, which can produce spatial frequency harmonics up to 4× the diffraction limit.^19^

Since resolution enhancement is restricted to one spatial dimension—along the modulated spatial direction—we developed a new imaging tomography to homogenize the resolution enhancement across the lateral plane. By rotating the illumination, or equivalently the object, the acquired signal can be expressed in a rotated frame as ${\tilde{S}}_{t,\phi }^{\left(q\right)}\;=\;{\langle u\left(y_{\phi }\right)c_{\phi }\left(x_{\phi },y_{\phi }\right)e^{-i2\pi q\kappa tx_{\phi }}\rangle }_{x_{\phi },y_{\phi }}$, where *x*_*ϕ*_ = cos *ϕ x* + sin *ϕ y* and *y*_*ϕ*_ = −sin *ϕ x* + cos *ϕ y*. The function *u*(*y*_*ϕ*_) determines the behavior of the projection, i.e., LT or FCT.

Lateral tomography is represented by uniform illumination along *y* (the direction perpendicular to the modulation direction, *x*) where we consider the case *u*(*y*_*ϕ*_) → 1. With this illumination pattern, we duplicate the formalism described in Ref. 15. Inserting the spatial frequency Fourier expansion of the object, $c_{\phi }\left(x_{\phi },y_{\phi }\right)\;=\;{\langle {\hat{C}}_{\phi }\left(f_{x_{\phi }},f_{y_{\phi }}\right)e^{-i2\pi \left(f_{x_{\phi }}x_{\phi }+f_{y_{\phi }}y_{\phi }\right)}\rangle }_{f_{x_{\phi }},f_{y_{\phi }}}$, and the rotated frame, *Ĉ*(*x*, *y*) → *Ĉ*_*ϕ*_(*x*_*ϕ*_, *y*_*ϕ*_), into the single sideband projection, we readily yield the Fourier slice theorem,

(1) $${\tilde{S}}_{t,\phi }^{\left(q\right)}\left(f_{x_{\phi }},f_{y_{\phi }}\right)\;=\;{\langle {\hat{C}}_{\phi }\left(qf_{x_{\phi }},f_{y_{\phi }}\right)\delta \left(f_{y_{\phi }}\right)\rangle }_{f_{y_{\phi }}}\;=\;{\hat{C}}_{\phi }\left(qf_{x_{\phi }},0\right),$$

after a few algebraic manipulations. Equation (1) describes a projection of the object spatial frequency distribution along the rotated coordinate system, $f_{x_{\phi }}\;=\;f_{x}(t)$, where the spatial frequency points are sampled as a spatial frequency-angle pair, as represented in Fig. 1(a). Equation (1) can also be written in the temporal frequency domain, ${\tilde{S}}_{v,\phi }^{\left(q\right)}\;=\;{\langle c_{\phi }\left(\frac{v}{q\kappa },y_{\phi }\right)\rangle }_{y_{\phi }}$, where it is clear that the LT is performing a spatial integral along *y*_*ϕ*_ as shown in the top row in Fig. 1(a).

Figure 1(a) illustrates the concept of LT data acquisition where a modulated light illumination pattern is used for the projection operator. The top row shows the spatial projection with an intensity modulation pattern at a particular time instance *t* and a spatial period given by $f_{x}^{-1}(t)$, while the bottom row shows the resulting spatial frequency support that is probed at a snapshot in time. In the backprojection algorithm, the spatial frequency support is summed together and a radial spatial frequency filter is applied, which is represented by the shaded gray radial ramp in the last column in the second row. The object is recovered with an inverse Fourier transform [see the last column in the first row in Fig. 1(a)]. Note that the formalism for CT can be recovered by substituting *y* → *z* and allowing *q* = 1.

Fourier Computed Tomography (FCT), by contrast, uses a line focus so that nonlinear optical processes can be driven efficiently by the illumination light. The line focus spatial distribution in *y*_*ϕ*_ is modeled, in the limiting case, as a Dirac-*δ* function, i.e., *u*(*y*_*ϕ*_) → *δ*(*y*_*ϕ*_). It is convenient to represent the time signal in the temporal frequency domain by taking the Fourier transform of the signal with respect to time, ${\tilde{S}}_{v,\phi }^{\left(q\right)}\;=\;{\langle u\left(y_{\phi }\right)c_{\phi }\left(x_{\phi },y_{\phi }\right)\delta \left(v-q\kappa x_{\phi }\right)\rangle }_{x_{\phi },y_{\phi }}$. Here, *ν* is the reciprocal variable for time. The projections and spatial integral produce an analogous spatial slice theorem, given by

(2) $${\tilde{S}}_{v,\phi }^{\left(q\right)}\left(\frac{x_{\phi }}{q},y_{\phi }\right)\;=\;{\langle c_{\phi }\left(\frac{x_{\phi }}{q},y_{\phi }\right)\delta \left(y_{\phi }\right)\rangle }_{y_{\phi }}\;=\;c_{\phi }\left(\frac{x_{\phi }}{q},0\right).$$

FCT is a limiting case of a thin spatial illumination where the spatial slice theorem is relevant. In the spatial slice theorem, ${\tilde{S}}_{v,\phi }^{\left(q\right)}$ is a projection in a rotated frame defined by $x_{\phi }=\frac{v}{\kappa }$. Finally, $c_{\phi }\left(\frac{x_{\phi }}{q},0\right)$ is a spatial slice of the object along *y*_*ϕ*_. The spatial slice is equivalent to performing the projection operation along the spatial frequencies perpendicular to the modulation direction, ${\tilde{S}}_{t,\phi }^{\left(q\right)}\;=\;{\langle {\hat{C}}_{\phi }\left(q\kappa t,f_{y_{\phi }}\right)\rangle }_{f_{y_{\phi }}}$; this is illustrated in bottom row of Fig. 1(b).

Once all the line images have been acquired with respect to *ϕ*, an FCT sinogram can be formed in the spatial frequency domain. The spatial frequency sinogram leads to a filtered spatial frequency backprojection algorithm which is a conjugate domain analog of the filtered backprojection algorithm.^1^ The *filtered spatial frequency backprojection algorithm* makes use of a radial spatial coordinate filter, rather than the radial spatial frequency filter employed in CT and LT. The filtered spatial frequency backprojection algorithm reconstructs images according to the formula

(3) $$\hat{C}\left(qf_{x},f_{y}\right)\;=\;\iint {\tilde{S}}_{v,\phi }^{\left(q\right)}e^{-i2\pi qf_{x_{\phi }}x_{\phi }}\left|x_{\phi }\right|\text{d}x_{\phi }\text{d}\phi ,$$

where *Ĉ*(*qf*_*x*_, *f*_*y*_) is the spatial frequency representation of the object, $e^{-i2\pi qf_{x_{\phi }}x_{\phi }}$ is the Fourier transform kernel in polar coordinates, and |*x*_*ϕ*_| is the radial spatial filter due to the Jacobian in the transformation from polar coordinates to Cartesian coordinates. A simple inverse Fourier transform recovers the 2D object in the spatial domain.

The object information probed with FCT is illustrated in Fig. 1(b), where the top row shows the spatial projections of the illumination onto the object and the bottom row is the resulting spatial frequency support that is probed with time. The spatial frequency support is summed together and the inverse Fourier transform is taken [see the bottom row last column of Fig. 1(b)]. In this case, a spatial radial filter is applied in real space to recover the object, as shown by the shaded gray radial ramp, in the top row’s last column of Fig. 1(b).

## III. EXPERIMENTAL SETUP

**FIG. 2.**
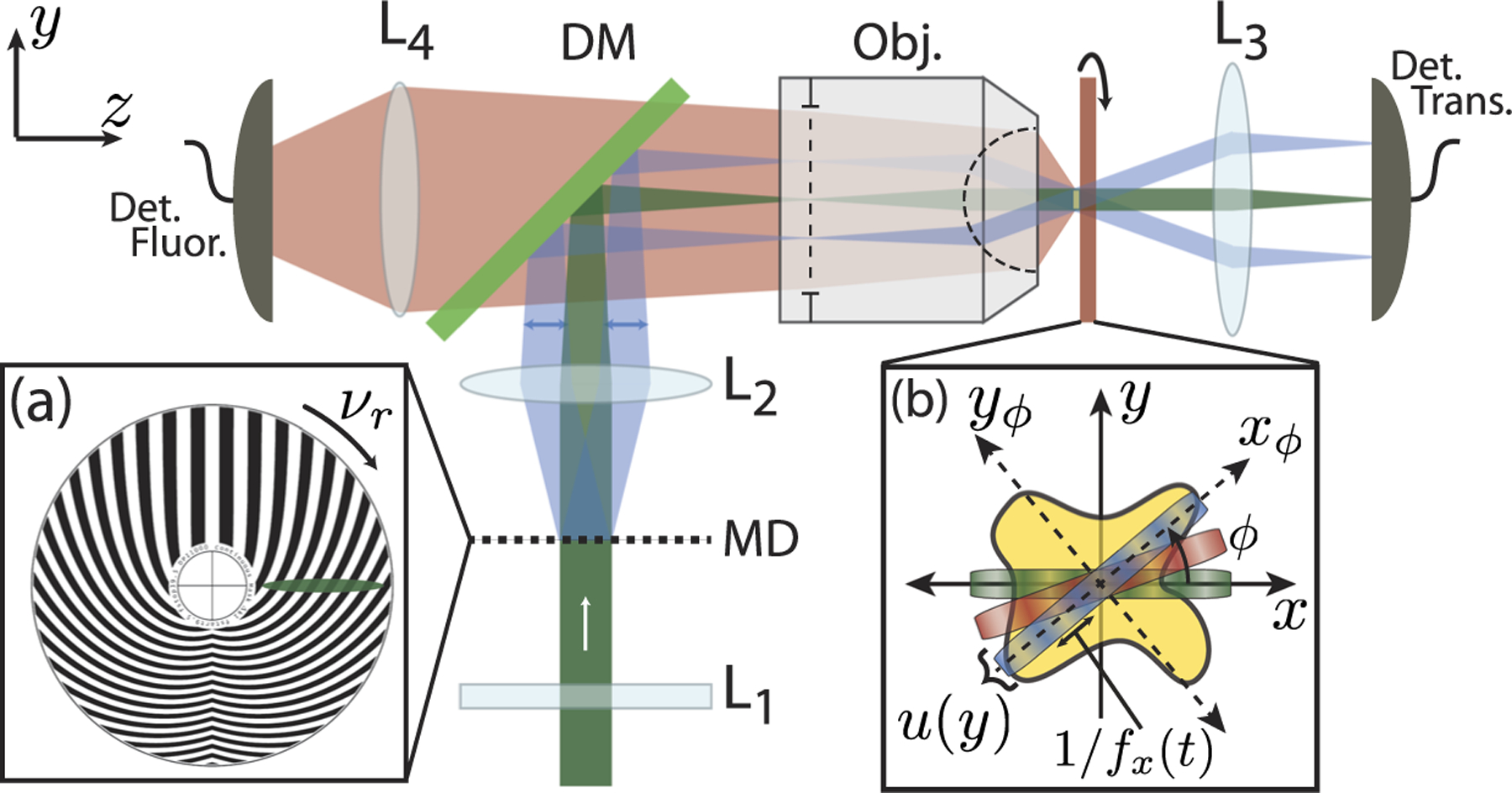
Panel (a) shows the FCT microscope schematic. The illumination beam is brought to a line focus on the modulator disk. The modulator disk [panel (a)] is image relayed to the sample. Both fluorescence (red beam) and absorption are collected by single pixel detectors. The sample is rotated on a rotation stage from [0, 2*π*] about the optic axis. Panel (b) shows the coordinate rotation of the beam with respect to the sample by an angle *ϕ*. *x*_*ϕ*_ and *y*_*ϕ*_ represent the rotated coordinate system. The various beam colors represent unique rotation angles with respect to the sample at a snapshot in time. *f*_*x*_(*t*) is the projected spatial frequency and *u*(*y*) is the intensity profile of the illumination beam in the *y* direction. *L*_1_: cylindrical lens, *L*_2–4_: spherical lenses, MD: modulator disc, DM: dichroic mirror, Obj.: objective lens, Det. Fluor.: fluorescence detector, Det. Trans.: transmission detector, and *ν*_*r*_: rotation frequency.

A schematic of the experimental setup is shown in Fig. 2(a). The specimen was illuminated by a spatially modulated *λ* = 532 nm wavelength continuous-wave (CW) laser (Lighthouse, Sprout). The spatial modulation on the line illumination is produced by bringing the illumination beam to a line focus onto a spinning modulator disk with a cylindrical lens.^16,17^ The modulated line was image relayed to the object plane with a 4-f imaging system constructed from lenses with focal lengths of 250 mm and 35 mm, respectively. The sample was mounted on a rotation stage (Newport PR50CC) to allow a full 360° rotation about the optic axis. Transmitted light was collected with a 0.25 NA aspheric lens and image relayed to a photodiode detector (Thorlabs DET100A). Fluorescent light emitted by the object was collected in the epidirection by relay imaging the object plane onto the surface of a PMT (Hamamatsu H9305). The fluorescent light was isolated using a dichroic beamsplitter (Semrock FF562-Di03) and an interference filter (Semrock FF01–593/40).

The 35 mm achromatic lens was chosen, instead of a typical high NA objective, to minimize the apparent effect of the axial and transverse wobble of the rotation stage as the object is rotated about the optic axis. Additionally, the back aperture of the objective lens was underfilled to increase the Rayleigh range so that the sample would stay in focus throughout the entire rotational scan, alleviating the defocus caused by the axial wobble. Since the objective lens was underfilled, the reconstructed image has a lower resolution than what would be allowed by the diffraction limit of the lens; however, this is a systematic limitation, and not one set by the physics or the reconstruction algorithm. The transverse wobble was 25–30 *μ*m over the rotation range; therefore, a correction protocol was adopted to mitigate this problem; the details are described in Sec. IV.

Figure 2(b), illustrates the illumination pattern at a snapshot in time. The green, red, and blue colors represent distinct rotation angles, *ϕ*, with respect to the sample. The width of the illumination, *u*(*y*), in our experimental setup was calculated to be ~3.87 *μ*m using the full width at half maximum (FWHM) of a fluorescence image. Using FWHM, the illumination numerical aperture (NA) was calculated to be ~0.07 and ~0.068, in *x* and *y*, respectively. The NA in *x* was calculated based on the largest crossing angle of the on axis beam and the scan beam, *θ*_*max*_ = 8.1.°

## IV. RESULTS AND DISCUSSION

While it is only necessary to scan from [0, *π*] radians to reconstruct an image, we scanned from [0, 2*π*] radians to help homogenize illumination beam inhomogeneities present in our current imaging system. We reconstructed each image with 360 uniformly spaced line images. In principle, only the line images are needed to perform an FCT reconstruction. However, the transverse wobble from the rotation stage caused the center of rotation to migrate resulting in an error in the image center. To correct this transverse wobble, and thus to enable the demonstration of the FCT imaging method, 2D images at every angle, *ϕ*, were acquired by scanning the line focus vertically.^20^ Every 2D image was numerically derotated by −*ϕ*. The derotated images were aligned in the *x* − *y* coordinates by maximizing the cross correlation between the images. The aligned images were then rotated back to their original rotation angle, *ϕ*. Finally, the center line image was extracted from the aligned 2D images, which formed the set of rotated line images required by spatial slice theorem. The image-centering protocol allowed us to correct transverse wobbles of the rotation stage. With the corrected line images, the FCT reconstruction using filtered spatial frequency backprojection in Eq. (3) was used to reconstruct 2D images of the object. This alignment procedure could be avoided by using a more precise rotation stage with less severe wobble.

**FIG. 3.**
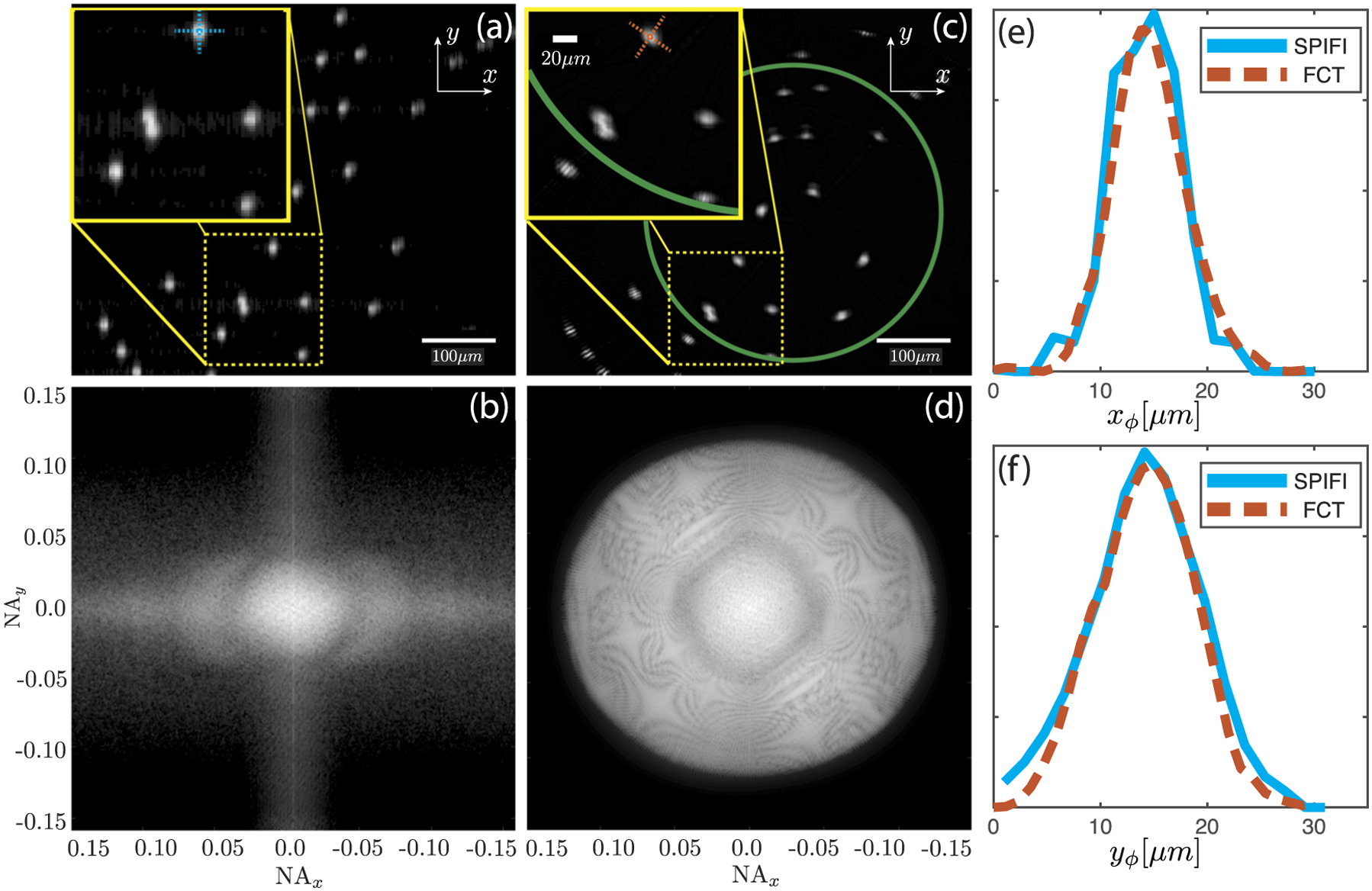
Panel (a) shows a second order SPIFI image of fluorescent stained 15 *μ*m polystyrene beads. The beads are elongated vertically because the resolution enhancement is only along the *x* coordinate. Panel (b) shows the Fourier transform of panel (a), and the *x* coordinate contains 2x higher spatial frequency support than the *y* coordinate. Panel (c) shows the second order FCT reconstruction of the fluorescent stained 15 *μ*m polystyrene beads. The yellow boxes indicate a zoomed-in region of the images to illustrate the resolution improvement between the two imaging types. The green circle shows the region where no radial sampling artifacts should appear according to the angular sampling criteria. Panel (d) shows the Fourier transform of panel (c); the spatial frequency support is isotropic. The sharp cutoff in the spatial frequency is due to the filtering used in the image reconstruction. Panels (e) and (f) show lineouts from both SPIFI and FCT images. The lineouts are taken along the *x*_*ϕ*_ and *y*_*ϕ*_ directions indicated by the colored dotted lines in panels (a) and (c).

FCT works with any contrast mechanism, however, we only present fluorescence results for brevity. Figures 3(a)–3(d) show a comparison between second order fluorescent SPIFI and second order FCT using 15 *μ*m fluorescent stained polystyrene beads (LifeTechnologies, FocalCheck Slide 1, Well A1). Figure 3(a) shows a second order SPIFI image. The yellow box shows a zoomed in region to better see the asymmetry in the resolution due the enhanced resolution in the *x* direction. Figure 3(b) shows the Fourier transform of Fig. 3(a). The frequency support shows the NA in the *x* coordinate that extends to 0.13 while the NA in the *y* coordinate extends to 0.068, which gives rise to the anisotropy in Fig. 3(a). Figure 3(c) shows the second order FCT reconstruction of the fluorescent stained beads. The yellow box shows a zoomed in region to better visualize the improvement in the resolution. Figure 3(d) shows the Fourier transform of Fig. 3(c). The frequency support extends to 0.13 NA isotropically. The dark ring is caused by the filtering applied in the FCT reconstruction.

Upon examining Fig. 3(c), it is evident that the images of the beads at a large radial coordinate appear elongated azimuthally. This appears due to the fact that our illumination beam line focus possesses a finite spatial frequency support along *y*_*ϕ*_, which produces a shift-variant distortion that grows with an increasing radius from the beam rotation point. The effect of this distortion will be reduced with both a higher NA focusing condition and when optical nonlinearities are driven during the imaging process as both will tighten the line focus such that it forms a better approximation to a delta distribution. Moreover, as FCT is linear, careful measurement of the illumination beam will allow deconvolution of the reconstructed image.

We also note that on the edge of the reconstructed image, there are azimuthal oscillations. This is a result of deficient angular coverage causing a void in the acquired information. This reconstruction artifact can be avoided by sampling the rotation angles more densely. The number of angular samples needed for complete angular coverage can be calculated by *N*_*ϕ*_ = *πr*/*δy*, where *r* is the radius from the center of rotation and *δy* is the FWHM of the illumination beam along *y*_*ϕ*_. To avoid the azimuthal oscillations with our 630 *μ*m field of view, we needed to sample *N*_*ϕ*_ = 268. Using the sampling formula where *N*_*ϕ*_ = 180 unique samples over a domain of *ϕ* ∈ [0, *π*], we find an artifact free field of view of ≈440 *μ*m, which is illustrated in Fig. 3(c) by the green circle.

## V. CONCLUSION

In this article, we derived and demonstrated a new form of tomography, called Fourier Computed Tomography (FCT), which operates in the conjugate spatial and spatial frequency domains as compared to conventional computed and lateral tomography. We showed mathematically that FCT is a conjugate analog to both Computed Tomography and Lateral Tomography. By controlling the shape of the illumination along *y* via *u*(*y*), we can change the imaging modality and subsequent reconstruction algorithm, that is, for *u*(*y*) → 1, Lateral Tomography applies and for *u*(*y*) → *δ*, Fourier computed tomography applies. In this article, we derived the spatial slice theorem and show that the equivalent sinogram in the spatial frequency domain leads to a filtered spatial frequency back-projection algorithm. We have operated this new tomography in both absorbing and fluorescent modes for second order enhancement of imaging resolution. Finally, we showed that FCT is capable of achieving nearly isotropic enhanced lateral resolution, mitigating the anisotropic spatial frequency support of spatial frequency-modulated imaging. While only three-beam spatial frequency illumination was reported, we also note that the FCT algorithm is general so that other line imaging techniques could be used, such as coherent holographic image reconstruction by phase transfer, to achieve holographic 3D volume information,^16^ or direct optical phase extraction for quantitative phase contrast.^17^

This new tomography is applicable to any computational imaging technique that forms images with line illumination and can combine anisotropic spatial resolution images together to form a nearly isotropic high resolution image. Additionally, this method opens a pathway to extending spatial frequency projection super resolution imaging from one dimension to an isotropic enhanced image of the object in the lateral plane.

Finally, we note that SFP imaging methods directly record and report aberrations in the imaging process in the form of a phase modulation of the recorded signal.^21^ This property of SFP imaging will be used in combination with generalized FCT imaging to permit super resolution imaging in complex specimens where phase distortions accumulated through refractive index variations in the tissue cause severe errors and degradation of super resolution imaging methods.^22^ With SFP-based FCT imaging, we will be able to record and correct these instrument and specimen induced aberrations because those aberrations are automatically encoded and can be removed in the reconstruction algorithm.

## References

[1] Devaney A, “A filtered backpropagation algorithm for diffraction tomography,” Ultrason. Imaging 4, 336–350 (1982). 6891131 10.1177/016173468200400404

[2] Kak A and Slaney M, Principles of Computerized Tomographic Imaging (SIAM, 2001).

[3] Sentenac A and Mertz J, “Unified description of three-dimensional optical diffraction microscopy: From transmission microscopy to optical coherence tomography: Tutorial,” J. Opt. Soc. Am. A 35, 748–754 (2018). 10.1364/JOSAA.35.000748 29726491

[4] Huang D, Swanson E, Lin C, Schuman J, Stinson W, Chang W, Hee M, Flotte T, Gregory K, Puliafito C , “Optical coherence tomography,” Science 254, 1178–1181 (1991). 1957169 10.1126/science.1957169 PMC4638169

[5] Haeberlé O, Belkebir K, Giovaninni H, and Sentenac A, “Tomographic diffractive microscopy: Basics, techniques and perspectives,” J. Mod. Opt 57, 686–699 (2010).

[6] Pustovit VN and Markel VA, “Propagation of diffuse light in a turbid medium with multiple spherical inhomogeneities,” Appl. Opt 43, 104–112 (2004). 14714650 10.1364/ao.43.000104

[7] Maire G, Girard J, Drsek F, Giovannini H, Talneau A, Belkebir K, Chaumet PC, and Sentenac A, “Experimental inversion of optical diffraction tomography data with a nonlinear algorithm in the multiple scattering regime,” J. Mod. Opt 57, 746–755 (2010).

[8] Cotte Y, Toy FM, Jourdain P, Pavillon N, Boss D, Magistretti PJ, Marquet P, and Depeursinge CD, “Marker-free phase nanoscopy,” Nat. Photonics 7, 113 (2013).

[9] Darrell A , “Weighted filtered backprojection for quantitative fluorescence optical projection tomography,” Phys. Med. Biol 53, 3863–3881 (2008). 18583727 10.1088/0031-9155/53/14/010

[10] Darrell A, Meyer H, Marias K, Brady M, and Ripoll J, “Weighted filtered backprojection for quantitative fluorescence optical projection tomography,” Phys. Med. Biol 53(14), 3863–3881 (2008). 18583727 10.1088/0031-9155/53/14/010

[11] Kim T, Zhou R, Mir M, Babacan DS, Carney SP, Goddard LL, and Popescu G, “White-light diffraction tomography of unlabelled live cells,” Nat. Photonics 8, 256 (2014).

[12] Choi W, Fang-Yen C, Badizadegan K, Oh S, Lue N, Dasari RR, and Feld MS, “Tomographic phase microscopy,” Nat. Methods 4, 717 (2007). 17694065 10.1038/nmeth1078

[13] Samelsohn G, “Transmission tomography of forward-scattering structures,” J. Opt. Soc. Am. A 33, 1181–1192 (2016). 10.1364/JOSAA.33.001181 27409448

[14] Hoover EE, Field JJ, Winters DG, Young MD, Chandler EV, Speirs JC, Lapenna JT, Kim SM, Ding S.-y., Bartels RA, Wang JW, and Squier JA, “Eliminating the scattering ambiguity in multifocal, multimodal, multiphoton imaging systems,” J. Biophotonics 5, 425–436 (2012). 22461190 10.1002/jbio.201100139 PMC3670971

[15] Schlup P, Futia G, and Bartels RA, “Lateral tomographic spatial frequency modulated imaging,” Appl. Phys. Lett 98, 211115 (2011).

[16] Field JJ, Winters DG, and Bartels RA, “Plane wave analysis of coherent holographic image reconstruction by phase transfer (CHIRPT),” J. Opt. Soc. Am. A 32, 2156–2168 (2015). 10.1364/JOSAA.32.002156 26560930

[17] Stockton PA, Field JJ, and Bartels RA, “Single pixel quantitative phase imaging with spatial frequency projections,” Methods 136, 24–34 (2018), methods in quantitative phase imaging in life science. 29107101 10.1016/j.ymeth.2017.10.007

[18] Field JJ, Winters DG, and Bartels RA, “Single-pixel fluorescent imaging with temporally labeled illumination patterns,” Optica 3, 971–974 (2016).

[19] Field JJ, Wernsing KW, Domingue SR, Motz AMA, DeLuca KF, DeLuca JG, Kuciauskas D, Levi DH, Squier JA, and Bartels RA, “Super-resolved multimodal multiphoton microscopy with spatial frequency-modulated imaging,” Proc. Natl. Acad. Sci. U. S. A 113, 6605–6610 (2016). 27231219 10.1073/pnas.1602811113 PMC4914181

[20] Futia G, Schlup P, Winters DG, and Bartels RA, “Spatially-chirped modulation imaging of absorbtion and fluorescent objects on single-element optical detector,” Opt. Express 19, 1626–1640 (2011). 21263702 10.1364/OE.19.001626

[21] Bartels RA and Field JJ, “Digital aberration correction of fluorescent images with coherent holographic image reconstruction by phase transfer (CHIRPT),” Proc SPIE 9713, 97130B (2016).

[22] Booth M, Andrade D, Burke D, Patton B, and Zurauskas M, “Aberrations and adaptive optics in super-resolution microscopy,” Microscopy 64, 251–261 (2015). 26124194 10.1093/jmicro/dfv033 PMC4711293

